# Tracking Visual Statistical Learning with Steady-State Visual Evoked Potentials: Effects of Exemplar and Category Information

**DOI:** 10.1162/OPMI.a.358

**Published:** 2026-06-17

**Authors:** Natasa Ganea, Dominik Garber, Richard N. Aslin, David J. Lewkowicz

**Affiliations:** Child Study Center, Yale School of Medicine, New Haven, CT, USA; Central European University, Vienna, Austria; Department of Psychology, Yale University, New Haven, CT, USA; Wu Tsai Institute, Yale University, New Haven, CT, USA

**Keywords:** statistical learning, EEG, SSVEP, neural entrainment

## Abstract

This study examined visual statistical learning using EEG-based steady-state visual evoked potentials (SSVEP). Fifty-one adults were exposed to image sequences organized into triplets across three conditions (*n* = 17 per condition) in which the alignment of category-level and exemplar-level information was manipulated. Neural entrainment at the triplet frequency (1.11 Hz) differed significantly across conditions (*η*_p_^2^ = .13), with stronger responses in the Single-Category and No-Category conditions than in the Mixed-Category condition. There were no differences at the image frequency (3.33 Hz; *η*_p_^2^ = .05). Behavioral reaction times mirrored this pattern, showing faster responses to the last exemplar in the triplet in the Single-Category (*η*_p_^2^ = .71) and No-Category (*η*_p_^2^ = .22) conditions, but not in the Mixed-Category (*η*_p_^2^ = .10) condition. Both signal-to-noise ratio (SNR) and inter-trial coherence (ITC) captured neural entrainment across fronto-central and parietal-occipital electrode clusters. These findings validate SSVEP as an online measure of visual statistical learning and demonstrate that category-exemplar mismatch interfered with statistical learning.

## INTRODUCTION

Extracting structural information from the rich stimulus environment in our everyday world requires powerful learning mechanisms. Although reinforcement learning, which has a long tradition in psychology and cognitive science, is one such mechanism (Dayan & Niv, 2008), another mechanism operates by mere exposure to stimuli without the benefit of causal attributions derived from reward. This sort of unsupervised mechanism, commonly known as statistical learning, emerged in the 1990s and is now recognized as a powerful contributor to learning in many domains, especially when contingent reward plays a less significant role than it does later in life (see Saffran & Kirkham, 2018, for a comprehensive review).

Much of what we know about statistical learning comes from behavioral exposure–test paradigms in which participants are first familiarized with a structured stimulus stream and then assessed with a pos*t*-test. In adults, post-tests commonly require explicit or metacognitive judgments (e.g., familiarity decisions in two-alternative forced choice tasks or rating tasks), whereas infant work typically relies on spontaneous looking measures (Saffran et al., 1996). Reaction time (RT) paradigms provide an alternative behavioral measure in children and adults by indexing faster responses to predictable than to unpredictable items within a statistically coherent chunk (e.g., item 3 vs. item 1 within learned triplets; Hunt & Aslin, 2001). These methodological traditions have been invaluable for establishing that unsupervised learning occurs, but they leave open a more specific theoretical question: which regularities are being encoded, and at what level of abstraction?

Many statistical learning paradigms define the to-be-learned structure at the level of individual exemplars (e.g., three specific syllables or images forming a triplet), such that successful learning requires encoding item-to-item transitions. However, naturalistic input affords multiple descriptions, and learners can extract regularities at different representational levels. Regularities may be encoded at the category level (i.e., members of one category predict members of another category) or at the exemplar level (i.e., one specific item predicts another item). For example, when exemplars from two categories are interleaved such that one category reliably follows another category, learners acquire predictive relationships that generalize beyond the trained items, consistent with category-level learning (e.g., Brady & Oliva, 2008; see also Jung et al., 2021, for similar findings in children). Conversely, when the input supports stable item-to-item transitions (i.e., fixed exemplar pairings), learners can form more detailed, exemplar-level predictive models, even when transitions cross category boundaries (Emberson & Rubinstein, 2016; see also Jun & Chong, 2018). These results suggest that learners can flexibly extract either category-level or exemplar-level regularities, depending on the structure of the input. However, other work indicates that category information can interact with (and sometimes reshape) learning of exemplar-level regularities (e.g., Emberson & Rubinstein, 2016).

One mechanism by which category information can impact statistical learning is through perceptual similarity among exemplars in the sequence of items, which can signal category membership and bias how learners segment the sequence into predictive chunks. Research shows that similarity within chunks (e.g., shared pitch or color) can facilitate statistical learning, and when similarity cues span a statistically defined chunk, learners can recover structure even in the presence of irrelevant intervening elements (Creel et al., 2004; Turk-Browne et al., 2005; Wang et al., 2020). Consistent with this facilitatory role of perceptual similarity, Rogers et al. (2021) found better learning when high-probability transitions linked two exemplars from the same perceptual category (within-category pairs) than when they linked exemplars from different categories (across-category pairs), suggesting that category membership can support the encoding of exemplar-to-exemplar transitions.

At the same time, perceptual similarity does not always facilitate learning of sequential regularities. Emberson and Rubinstein (2016, Exp. 4) presented streams in which stable object-level pairings (e.g., bird → dog) were instantiated using varying viewpoints of each object (e.g., bird₁→dog₁, bird₂→dog₂). Although the object-level pairing was consistent, at test participants showed little evidence of learning viewpoint-specific mappings, failing to reliably distinguish familiar from novel pairings (e.g., bird₁→dog₁ vs. bird₁→dog₂). Emberson and Rubinstein interpreted this pattern as reflecting learners’ prior expectations: it is common for objects to predict other objects, but less common that a particular viewpoint of an object predicts a particular viewpoint of another object. An alternative interpretation of the results is that perceptual similarity encouraged an object-level organization of the visual stream that competed with encoding the intended viewpoint-to-viewpoint transitions, thereby reducing sensitivity to exemplar-level regularities.

One way to reconcile these mixed findings is to treat category information as a cue that can yield both facilitation or interference depending on its alignment with statistically defined chunks. This logic can be illustrated with a simple analogy. If a learner repeatedly sees different animals, each performing a sequence of actions, the stream encourages an exemplar-level model in which predictions are tied to each animal (e.g., Chunk 1: dog standing → dog yawning → dog sleeping; Chunk 2: monkey walking → monkey climbing → monkey jumping; Chunk 3: cat eating → cat sitting → cat stretching). In this case, the learner forms a detailed model of the sequence of actions each animal performs. By contrast, if a stable category order repeats across chunks, then a more abstract category-level model becomes sufficient (e.g., Chunk 1: dog standing → monkey climbing → cat stretching; Chunk 2: dog yawning → monkey jumping → cat eating; Chunk 3: dog sleeping → monkey walking → cat sitting). In this latter case, the learner can adopt the category-order rule “dog → monkey → cat” as a positional regularity, deemphasizing information about the animals’ actions. Critically, prior work has rarely been designed to test both outcomes within the same paradigm: category structure may facilitate learning when aligned with statistically defined chunks but interfere with learning when it supports an alternative chunking of the stimuli. Moreover, aligned category cues can act as salient boundary markers, raising the additional question of whether category cues and statistical cues have an additive effect on the speed or strength of chunk learning. Disentangling these possibilities requires a measure that tracks learning during the exposure phase, rather than relying solely on pos*t*-test behavior, motivating the use of neural entrainment approaches.

Online neural measures have been developed to assess the rate of statistical learning without requiring an explicit behavioral response. Batterink and Paller (2017) combined speech stimuli (like those used by Saffran et al., 1996) with an EEG measure of neural entrainment commonly used in research on visual processing. This measure, known as the steady-state visual evoked potential (SSVEP), reflects the brain’s response to a stimulus presented repeatedly at a fixed frequency (e.g., 6 Hz; see Norcia et al., 2015, for a review). In typical SSVEP studies, researchers examine the frequency spectrum of the EEG signal for increased power at the stimulation frequency (the baserate) and compare it to neighboring frequencies serving as controls. When higher-order structure is embedded at a slower periodicity (e.g., every third item, corresponding to 2 Hz), the brain may also show enhanced power at this lower sub-baserate frequency, reflecting sensitivity to the two-level hierarchy of information structure (Liu-Shuang et al., 2016; Rossion et al., 2015). Using this approach, Batterink and Paller found that, in adults, neural entrainment at the triplet frequency reliably reflected sensitivity to learned statistical regularities, and it correlated with behavioral pos*t*-test performance (see also Batterink et al., 2024; Batterink & Paller, 2019). Entrainment approaches have since been extended to infants (see Kabdebon et al., 2022; Peykarjou, 2022 for reviews), providing evidence for sensitivity to higher-order structure in both visual and auditory domains (e.g., Batterink & Choi, 2021; Calce et al., 2024; Capparini et al., 2026; Choi et al., 2020; de Heering & Rossion, 2015).

Despite robust evidence for neural entrainment to statistically defined chunks in auditory paradigms, visual statistical learning has been less explored with non-invasive EEG. Intracranial work (ECoG) provides compelling evidence of entrainment to pair-based visual structure and shows that entrainment strengthens with exposure (Sherman et al., 2023). These intracranial studies typically report localized entrainment at modality-relevant posterior regions for visual streams (e.g., occipital and parietal cortex), with additional involvement of the frontal and temporal areas often implicated in domain-general sequence processing (Henin et al., 2021; Sherman et al., 2022, 2023). Scalp EEG evidence for entrainment to triplet structure in visually presented streams is rare; to our knowledge, only one study has used scalp EEG to measure entrainment to triplets in a visually presented stream of written syllables (Sáringer et al., 2024). Importantly, because those stimuli had minimal shared visual features, this approach does not address how perceptual similarity and category membership modulate visual statistical learning.

The present study aimed to test how category information shapes learning of statistically defined visual triplets by manipulating the alignment between category membership and triplet structure across three conditions and measuring neural entrainment at the triplet frequency. In the Single-Category condition, all three items within a triplet belonged to the same category, and each triplet came from a different category. Thus, category membership was aligned with triplet boundaries, so boundaries were marked both by statistical cues (low transitional probabilities between triplets) and by an additional perceptual/category cue (category change), allowing the possibility of additive cueing (faster/stronger learning). In the No-Category condition, exemplars came from 12 different categories, removing systematic category–boundary alignment such that triplet boundaries were defined by statistical structure alone. Finally, in the Mixed-Category condition, four categories were used, as in the Single-Category condition, but now each triplet contained one exemplar from three of the four categories in no fixed order. As a result, salient category information was present, but it was distributed across triplets, thereby testing whether misaligned category structure interfered with exemplar-defined chunks.

We made three predictions about the interaction between category-level and exemplar-level information: (1) if category information plays no role, entrainment at the triplet frequency should be equivalent across conditions; (2) if aligned category cues provide an additive benefit, triplet-frequency entrainment should be higher and/or emerge more rapidly in the Single-Category than the No-Category condition (Single-Category > No-Category); and (3) if misaligned category information competes with chunk formation, entrainment should be higher in the No-Category than the Mixed-Category condition (No-Category > Mixed-Category).

## METHOD

### Pre-Registration and Data Availability

This experiment was not pre-registered. Stimuli, data, and custom R and MATLAB scripts are available on OSF: https://osf.io/mhr6y/?view_only=8879b507ac9644c384623f1260dc90d8.

### Participants

Sixty-four adults (34 female) aged between 18 and 46 years old (*M* = 26.97, *SD* = 6.33) participated in the study. Data from 13 participants were excluded from the final analysis because they fell asleep during the learning phase (*n* = 2), had fewer than 20 artifact-free EEG epochs (*n* = 4), produced excessive blinking (*n* = 1), or because the equipment failed, resulting in data loss or inaccurate stimulus timing (*n* = 6). The remaining 51 participants had normal or corrected to normal vision and had no history of psychiatric or neurological disorders. Thirty participants identified as White, 17 as Asian, 2 as Black, and 2 as Mixed Race. Participants provided signed informed consent and were compensated $25 for participating in the experiment. The study protocol was approved by the university’s Institutional Review Board.

A priori power analysis was conducted using G*Power 3.1 to determine the required sample size for detecting an interaction effect of Condition and Frequency on SNR, using a 2 × 3 ANOVA with Condition as a between-subjects factor (*Single-Category, No-Category, Mixed-Category*) and Frequency as a within-subjects factor (*1.11 Hz – triplet frequency, 3.33 Hz – image frequency*). Although all three conditions in our design involved structured input, they differed in how readily participants could extract regularities due to factors such as perceptual similarity between exemplars and category alignment. Nonetheless, we expected to obtain evidence of chunk learning at the triplet frequency of 1.11 Hz and predicted that we would obtain a Condition effect at this frequency. We based our effect size estimate on prior research showing robust differences in neural entrainment (measured through inter-trial phase coherence) in response to structured versus random auditory sequences (Batterink & Paller, 2017; Sáringer et al., 2024). Although these studies compared structured versus unstructured input, we reasoned that any differences in statistical learning across our three structured conditions – particularly between the *Single-Category* and the *Mixed-Category* conditions – may yield similarly strong effects. To remain conservative, we based our analysis on a medium effect size of *η*_p_^2^ = 0.06 (Cohen’s *f* = 0.25), with *α* = 0.05 and desired power (1 − *β*) = 0.80. The power analysis indicated that a minimum total sample size of 42 participants (i.e., 14 per condition) would be required. To account for potential EEG data loss due to artifact rejection, we aimed to slightly oversample, targeting a final sample of 51 participants (i.e., 17 per condition).

### Stimuli and Apparatus

The total stimulus set consisted of 20 cartoon images from the Freepik image bank (https://www.freepik.com/). Each participant viewed a subset of 12 of these images depending on the experimental condition to which they were assigned. The images measured 7 cm × 7 cm and appeared on a grey background (RGB: 128, 128, 128), in the center of a 24-inch LCD monitor (resolution: 1920 × 1080; dimensions: 52.5 cm × 29.5 cm; refresh rate: 60 Hz; refresh cycle duration = 16.67 ms). The cartoons subtended 6.16 × 6.16 degrees of visual angle from the participants’ viewing distance of 65 cm.

We conducted the experiment on a PC (Intel i5-10500 CPU 3.10GHz, RAM 16 GB) running Windows 10, Matlab R2022b, and Psychtoolbox 3.0.18 (Brainard, 1997; Kleiner et al., 2007; Pelli, 1997). Custom Matlab scripts controlled the stimulus presentation and the recording of EEG and eye-tracking data. We used a photodiode (StimTracker Duo Cedrus, San Pedro, CA) to ensure that the stimuli were presented at the appropriate temporal intervals. The photodiode was affixed to the bottom left of the monitor and sent a trigger to the EEG amplifier whenever it detected a change from black to white of a small square that accompanied the presentation of each new image (i.e., every 300 ± 3 ms during the learning trials).

Eye gaze position was recorded continuously with an SR Eyelink 1,000 eye-tracker running at a sampling rate of 500 Hz and equipped with a desktop mount infrared illuminator TT-890 nm and a 16 mm lens (SR Research, Ottawa, Canada). The eye-tracker was located on a desk between the participant and the stimulus-presentation monitor, and it tracked the participant’s right eye (the distance between eye and eye-tracker was 54.6 cm). To reduce data loss, participants positioned their heads on a chin rest located 65 cm away from the monitor. EEG data were collected continuously using a 128-electrode HydroCel Geodesic Sensor Net (HydroCel GSN; Megastim EGI, Eugene, OR). The EEG signals were amplified with an EGI amplifier (amp NA300) and digitized at a sampling rate of 500 Hz (software: NetStation 5, Megastim EGI). Impedances were kept below 50 kΩ for all the electrodes and the data were referenced online to the vertex electrode (Cz) and re-referenced offline to the average of all the electrodes.

### Procedure and Design

**Figure 1.**
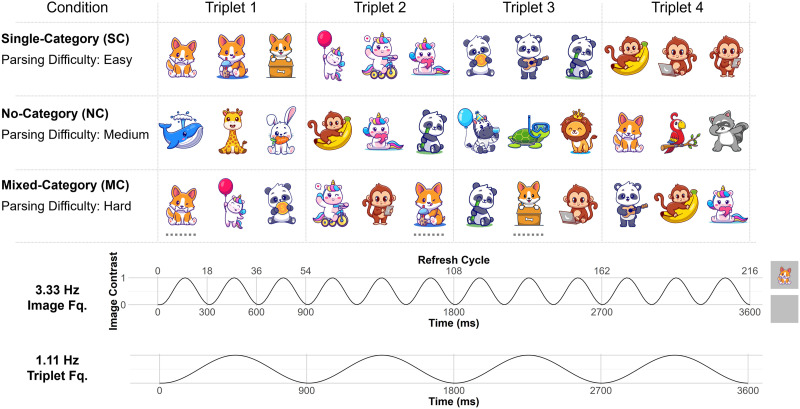
Stimuli presented during the experiment and learning trial structure. *Note*. During the learning phase, participants viewed 12 cartoon animal images (Freepik; https://www.freepik.com/) organized into four distinct triplets. Within each triplet, images appeared in a fixed order (within-triplet transitional probability = 1), while triplets were presented in a pseudo-random sequence, avoiding consecutive repeats (across-triplet transitional probability = 0.33). Images were displayed every 300 ms (3.33 Hz), and triplets occurred every 900 ms (1.11 Hz), with image contrast modulated sinusoidally. Participants were assigned to one of three learning conditions. In the Single-Category condition, each triplet consisted of visually similar images from the same animal category, providing a strong cue for parsing. In the No-Category condition, images came from 12 different categories, minimizing visual overlap but offering no category cue to triplet structure. In the Mixed-Category condition, images were drawn from the same four animal categories as in the Single-Category condition; however, each triplet contained exemplars from multiple categories, leading to greater visual interference across triplets and increased working memory demands. In both the No-Category and Mixed Category conditions, images were randomly grouped across participants. Participants completed 14 learning trials (28.8 seconds each), with short self-paced intervals between trials featuring a central animation (5.5 cm × 5.5 cm; minimum 1 s).

The experiment consisted of a learning phase (14 trials) and a pos*t*-test phase (36 trials). During each learning-phase trial, participants viewed a sequence of 96 images consisting of 4 distinct triplets, with each triplet repeated 8 times during the sequence. Within each triplet, the images always appeared in the same fixed order (i.e., Image 1, Image 2, Image 3) but, to ensure that the same triplet was never shown consecutively, the triplets were presented in a pseudo-random order. For instance, Triplet 1 could be followed by Triplet 2, 3, or 4, but not by another instance of Triplet 1. This presentation format ensured an image-to-image transitional probability of 1 within a triplet and a probability of 0.33 across different triplets. As can be seen in Figure 1, the images appeared at a frequency of 3.33 Hz (i.e., every 300 ms or 18 monitor refresh cycles) and became gradually visible and invisible in an approximate sinusoidal modulation of image contrast. Each learning trial lasted 28.8 seconds and began and ended with a 900 ms interval (i.e., one triplet) when the stimuli gradually faded in/out without reaching maximal contrast depth. We employed this gradual fading in/out to minimize eye fatigue and blinking caused by sudden stimulus onset/offset. Learning trials were excluded from the analysis if participants failed to look at the stimuli for more than 10 seconds. Every learning trial was separated by a minimum 1 s inter-trial interval during which a short animation of some geometric shapes converging in the center of the screen could be seen (5.5 cm × 5.5 cm). The start of each trial was controlled by the participants with a press of the spacebar.

To investigate the effects of sequence structure on learning, we employed a between-subjects design defined by 3 conditions: *Single-Category, No-Category*, and *Mixed-Category* Triplets. In the Single-Category condition, each triplet consisted of visually similar images from the same animal category (e.g., three bears), and each of the four triplets was associated with a different category. Thus, category membership was perfectly correlated with triplet structure, providing a strong cue for parsing the sequence. In the No-Category condition, exemplars were drawn from 12 different animal categories, ensuring that there was no correlation between category membership and triplet structure. In this condition, participants had to rely solely on the transitional probabilities between individual images to learn the sequence. Finally, in the Mixed-Category condition, the images were drawn from four animal categories except that the categories were distributed across the four triplets. Specifically, each triplet contained exemplars from three of the four categories and exemplars from the same category appeared in multiple triplets. This design feature of the Mixed-Category condition introduced potential interference between category-level and exemplar-level information, making the sequence harder to parse and the triplets more difficult to learn. In both the No-Category and Mixed-Category conditions, images were assigned randomly across participants.

The post-test phase assessed participants’ learning of the 4 triplets. Each trial presented a shorter segment (i.e., 12 images) of the visual sequence shown during the learning phase. This segment included the 4 triplets, with each triplet appearing once. Participants’ task was to press the spacebar when they detected a target image within the sequence. The target was one of the 12 images seen during the learning phase and the same target was used across 3 test trials. The target could appear in the first, second, or third position of its triplet. Critically, it always maintained this original ordinal position from the learning phase and was embedded within one of the two triplets positioned in the middle of the 12-image sequence. We did not want the target image to be in the first triplet to avoid stimulus onset effects that would interfere with rapid reaction times. We also did not want the final triplet to contain the target because it could lead to faster reaction times (e.g., by a process of elimination given that the first three triplets did not contain the target).

**Figure 2.**
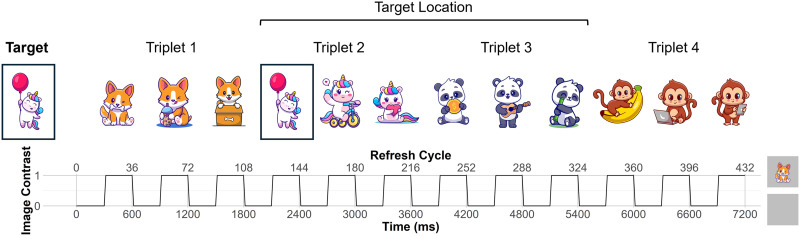
Post-test trial structure. *Note*. During the post-test phase, participants viewed shorter sequences of 12 images depicting the four triplets from the learning phase. At the start of each trial, a target image (one of the 12 images) was displayed, and participants pressed the spacebar when they detected it during the sequence. Images were presented for 300 ms each, with a 300 ms gap between them, using an on/off contrast modulation. The target appeared within one of the two middle triplets, and participants had 1 s to respond; early or late responses were counted as incorrect. There were 36 test trials (12 targets × 3 repetitions), each lasting 7.2 seconds. During the inter-trial interval, participants viewed a short central animation (5.5 cm × 5.5 cm; minimum duration: 1 s) and pressed the spacebar to begin the next trial.

As can be seen in Figure 2, the images in the post-test sequence were presented for 300 ms each, with a 300 ms inter-stimulus interval to allow participants to respond. Unlike in the learning trials, image contrast in the post-test trials was modulated in a square-wave fashion, producing a distinct on/off stimulus presentation. This approach was intended to reduce variability in perceived image onset (which can occur with gradual contrast increases) and to establish a precise starting point for measuring reaction times. At the start of each post-test trial, participants viewed the target image and pressed the spacebar when ready to begin the trial. Each post-test trial lasted 7.2 s, and participants had 1 s to press the spacebar from target onset to respond. Anticipatory or late responses were marked as inaccurate, and no feedback was provided.

The experiment was conducted in a sound-attenuated, dimly lit room. Participants were informed that the experiment consisted of two phases: a learning phase, during which they would watch a sequence of images, and a post-test phase, during which they would view a shorter version of the same sequence. They were also told that a target image would be displayed at the beginning of each post-test trial and that their task was to press the spacebar within 1 s of detecting the target image in the sequence of images. Once participants understood the instructions, they were fitted with a HydroCel GSN EEG cap, ensuring that all the electrodes made good contact with the scalp and that their impedance was below 50 kΩ. Then, we administered a 5-point eye-tracker calibration routine (top, bottom, left, right, and central screen positions) during which the calibration points were presented in a random sequence against a grey background. The calibration points remained on the screen until the system confirmed a stable eye fixation. The eye-tracker calibration was deemed successful if the average calibration error was below 2 degrees of visual angle. If the average error exceeded this threshold, the calibration procedure was repeated.

After the eye-tracker calibration, participants were reminded of the instructions for the learning trials and were told to start the experiment by pressing the spacebar when ready. Before each trial, participants had to look at a central point on the screen and the eye-tracker measured any deviation from the expected gaze position (i.e., drift check). If the drift error exceeded 0.5 degrees of visual angle, the calibration procedure was repeated. Once acceptable re-calibration was confirmed, the experiment continued from the trial where it was interrupted. Once participants completed the 14 learning trials, they were given a short break (approximately 2 min), were reminded of the instructions for the 36 post-test trials and asked to press the spacebar when they were ready to proceed.

Key presses, eye-movements, and EEG data were recorded throughout the experiment. To ensure that the EEG and eye-tracking data were synchronized, the MATLAB script that controlled the presentation of the stimuli sent one trigger to the eye-tracking computer and another trigger to the EEG computer within a 2-ms time-window at the onset and offset of each trial. The experiment lasted approximately 25 minutes, including the eye-tracker calibration procedure. At the end of the experiment, the EEG cap was removed, and participants were debriefed. The data were collected between May and November 2023.

### Data Processing

The eye-tracking data were exported using Eyelink Data Viewer (SR Research). Saccades and fixations were identified using the default Eyelink settings based on velocity and acceleration. Fixation durations within a predefined region of interest (ROI) surrounding the image were calculated and a report summarizing the total dwell time and average pupil size per trial was generated. The list of events/triggers detected by the EGI amp NA300 was processed in MATLAB to assess the precision of stimulus presentation during the learning trials and to remove the 900 ms fade-in/out interval at the beginning and end of each learning trial. The remaining 27 s of the learning trial were divided into three 9 s EEG epochs. The decision to split the learning trials into smaller EEG epochs was made to improve data retention. In a preliminary data analysis using 27 s EEG epochs, the automatic EEG artifact rejection tool excluded too many epochs due to movement artifacts detected at various points during the trial. To increase data retention while excluding noisy EEG segments, we split the data into non-overlapping 9 s EEG epochs that corresponded to 10 triplets.

**Figure 3.**
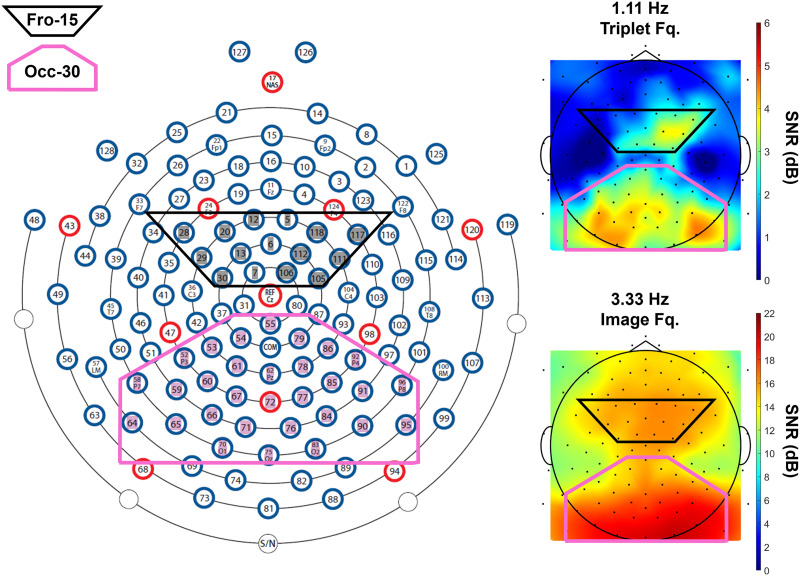
Clusters of electrodes used for the statistical analysis of the EEG data, and mean SNR at 1.11 Hz and 3.33 Hz pooled across experimental conditions. *Note*. Separate clusters of 15 frontal and 30 occipital electrodes were used for the statistical analysis of the EEG data. The topo plots represent the signal-to-noise ratio (SNR) in decibels (dB) at 1.11 Hz (triplet frequency) and 3.33 Hz (image frequency) averaged across all participants, irrespective of the experimental condition. SNR was stronger at the image frequency than the triplet frequency. Warmer colors represent higher SNR.

The primary goal of the current study was to investigate the brain mechanisms underlying visual statistical learning. Therefore, we only processed the EEG data from the learning trials, and we used the post-test trial data to determine whether the participants showed behavioral evidence of learning the triplets. EEG data processing was performed using the HAPPE+ER pipeline (Monachino et al., 2022; www.plasticityinneurodevelopmentlab.com/happeer), Letswave 6 (www.letswave.org/), and MATLAB. We used HAPPE+ER v4 to: (1) remove the 60 Hz line noise, (2) perform artifact correction using a soft wavelet threshold, (3) reject bad channels, (4) apply a bandpass FIR filter (0.3–40 Hz), (5) segment the EEG data into 9 s epochs, (6) reject epochs with at least one channel that crossed the amplitude threshold of ±150 *μ*V, (7) interpolate bad channels using a spherical interpolation, and (8) re-reference the EEG data to the average of all the channels. The re-referenced EEG epoch data were then imported into Letswave 6 and processed further by: (9) creating a cluster of 15 frontal electrodes and another cluster of 30 occipital electrodes (see Figure 3), (10) averaging the EEG data across unrejected epochs in the time domain, (11) applying the Fast Fourier Transformation (FFT) to extract the power spectral density, and (12) calculating the signal-to-noise ratio (SNR) on the extracted power. Bad channels were identified based on the default HAPPE+ER criteria: (a) the channel exhibited a flat line for more than 5 s, (b) the average power of the channel was more than 3.5 *SD* above or 5 *SD* below the average power of all the other channels, (c) the line noise/neural signal ratio for the channel was more than 6 SD away from the mean ratio across all the channels, and (d) the correlation between a particular channel and all the other channels was less than 0.8 (see Monachino et al., 2022).

Crucially, we chose the frontal and occipital electrode clusters as the primary source of data for the current study for two reasons. First, Sáringer et al. (2024) reported that the primary brain response to visual triplets was in the fronto-central and parieto-occipital electrodes (see also Henin et al., 2021; Sherman et al., 2023). Second, we found that the grand average SNR across all the participants (irrespective of the experimental condition) was highest in these two clusters of electrodes when compared to other brain areas at both 1.11 Hz and 3.33 Hz (see Figure 3).

SNR was calculated based on the formula (see Meigen & Bach, 1999):

$${SNR}_{dB}=10{\text{log}}_{10}\left(\frac{P_{\text{signal}}}{P_{\text{noise}}}\right)$$

where *P*_*signal*_ represents the power at the target frequency 1.11 Hz or 3.33 Hz) and *P*_*noise*_ is the average power in the 3 frequency bins on either side of the target frequency excluding the immediately adjacent bin. For example, for 3.33 Hz, *P*_*noise*_ was calculated from 2.89, 3.00, 3.11, 3.55, 3.66, and 3.77 Hz (excluded bins: 3.22 and 3.44 Hz). We chose three frequency bins on either side of target frequency (totalling 0.33 Hz per side) because our spectral resolution was 0.11 Hz (i.e., $\text{frequency}\;bin\;\text{width}=\frac{1}{\text{epoch duration}\;\left(s\right)}$ $=\frac{1}{9}$) and previous SSVEP studies used neighboring frequency bins that corresponded to a frequency range of ±0.2 Hz to ±0.7 Hz (see Buiatti et al., 2009; Liu-Shuang et al., 2022; Rossion et al., 2012). Higher SNR values represent more power at the target frequency than at the adjacent frequencies.

Finally, we calculated the inter-trial phase coherence (ITC; also known as phase-lock value) at 1.11 Hz and 3.33 Hz based on the formula (see Benjamin et al., 2021; Delorme & Makeig, 2004; Kabdebon et al., 2015):

$$ITC\left(f\right)=\left|\frac{1}{N}\sum_{n=1}^{N}\frac{X_{n}\left(f\right)}{\left|X_{n}\left(f\right)\right|}\right|$$

where *N* is the number of trials and *X*_*n*_(*f*) is the Fast Fourier transform of EEG epoch *n* at the frequency *f*. Dividing the Fourier transform by its absolute value provides the phase information at frequency *f*. The ITC was the average phase consistency across all the epochs. An ITC value of 1 indicates perfect phase alignment across all the epochs, while an ITC value of 0 indicates random phase alignment. Given that ITC and SNR are mathematically related (Van Diepen & Mazaheri, 2018; Victor & Mast, 1991), we only report the SNR analysis and the relationship between SNR and ITC in the results section (see Supplementary Materials for the complete ITC analysis). We opted to conduct both SNR and ITC analyses because a recent study that measured covert and overt attention in adults found that SNR was generally a more reliable index of attention than ITC (Ganea et al., 2025). Furthermore, Pinto et al. (2022) found ITC to be a relatively poor measure of individual differences in an auditory statistical learning study. We also analyzed the relationship between SNR and participants’ pos*t*-test reaction times and report that in Supplementary Materials.

## RESULTS

### Post-Test Trials

We analyzed participants’ accuracy and reaction time (RT) to the target stimulus during the pos*t*-test trials to assess learning and task compliance, using ANOVAs. For each ANOVA, we first tested the assumption of sphericity with Mauchly's test. When this assumption was violated, we applied a Greenhouse–Geisser correction to adjust the degrees of freedom (resulting in non-integer values).

#### Accuracy.

Participants’ accuracy was calculated by dividing the number of trials when the target image was correctly detected by the number of all the pos*t*-test trials. Mean accuracy was 0.97 (*SD* = 0.04), and the effect of Condition was marginally significant, *F*(2, 48) = 3.01, *p* = .059, *η*_p_^2^ = .11. Participants in the *Single-Category* condition (*M* = 0.96, *SD* = 0.06) were less accurate than those in the *No-Category* (*M* = 0.98, *SD* = 0.03) and *Mixed-Category* (*M* = 0.98, *SD* = 0.02) conditions, however the differences did not survive the Holm correction for pairwise comparisons (all *t’s* < 2.12, all *p’s* > .116).

#### RT.

Mean RT (in seconds) for correctly answered trials was calculated separately for each image in the triplet. As expected, the effect of image position within the triplet was statistically significant, *F*(1.27, 63.49) = 17.57, *p* < .001, *η*_p_^2^ = .26. Overall, across the three conditions, participants took progressively less time to respond across the triplet images, with the longest response times for *Image_1* (*M* = 0.39, *SD* = 0.04), followed by *Image_2* (*M* = 0.35, *SD* = 0.05), and the shortest for *Image_3* (*M* = 0.33, *SD* = 0.07; Holm-corrected pairwise comparisons: all *t’s* > 2.53, all *p’s* < .015). Crucially, this overall response time difference as a function of image position depended on the type of sequence participants learned, as indicated by a significant Image Position × Condition interaction in a two-way ANOVA (Image Position: within-subjects; Condition: between-subjects), *F*(3.24, 77.87) = 27.54, *p* < .001, *η*_p_^2^ = .53.

**Figure 4.**
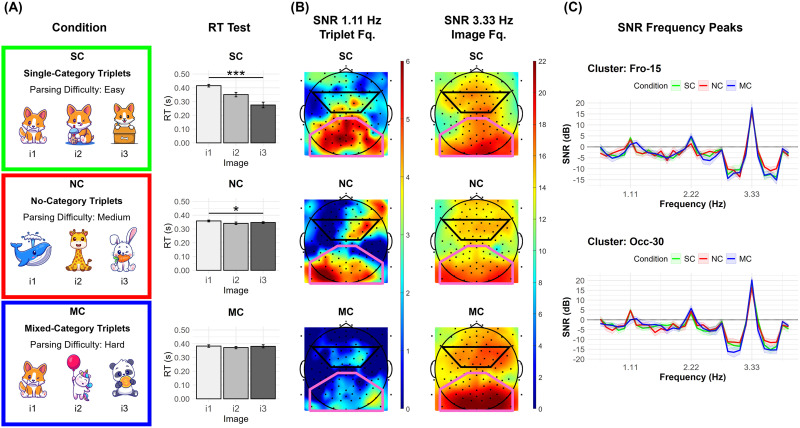
Mean RT for each image in the triplet in the post-test trials, and mean SNR at 1.11 Hz (triplet frequency) and 3.33 Hz (image frequency) in the learning trials. *Note*. (A) Bar plots represent mean RT to each image in the triplet, measured during the Post-test Trials, and split across experimental conditions. Error bars represent standard error of the mean. There was a significant difference in RT between Image 1 and Image 3 in the Single-Category (SC) condition and No-Category (NC) condition, but not in the Mixed-Category (MC) condition. (B) Topo plots represent mean SNR (in decibels) at 1.11 Hz (triplet frequency) and 3.33 Hz (image frequency) measured during the Learning Trials. SNR was stronger at the image frequency than the triplet frequency. Warmer colors represent higher SNR. (C) Line plots represent the mean SNR (in decibels), with shaded areas indicating the standard error of the mean for the frontal electrode cluster (top) and the occipital cluster (bottom). SNR peaked at 1.11 Hz, 3.33 Hz, and their harmonics. The grey horizontal line represents an SNR of 0 dB, meaning signal and noise are equal. Peaks above this line indicate that the power at a given frequency is higher than the power at adjacent frequencies.

To follow up the Image Position × Condition interaction, we conducted three separate one-way ANOVAs, one for each experimental condition. In the *Single-Category* condition, the effect of image position was statistically significant, *F*(1.46, 23.41) = 38.61, *p* < .001, *η*_p_^2^ = .71, with RT decreasing from *Image_1* to *Image_2* and from *Image_2* to *Image_3* (all *t* > 5.08, all *p* < .001; see Figure 4). In the *No-Category* condition, participants showed a similar RT pattern to the *Single-Category* condition, *F*(1.42, 22.75) = 4.54, *p* = .032, *η*_p_^2^ = .22, but only the difference between *Image_1* and the other two images remained significant after Holm correction (all *t’s* > 2.57, all *p’s* < .041). In contrast to the other two conditions, the effect of Image Position was not significant in the *Mixed-Category* condition, *F*(1.47, 23.53) = 1.77, *p* = .196, *η*_p_^2^ = .10, indicating that RTs did not differ in this condition as a function of the target’s position in the triplet.

### Learning Trials

The differential effects of Condition on RTs during the pos*t*-test trials suggested potential differences between conditions during the learning phase. To explore this possibility, we conducted separate analyses of the learning trials data by examining: (1) looking time to the stimuli, (2) SNR at 1.11 Hz (triplet frequency) and 3.33 Hz (image frequency), and (3) the relationship between SNR and ITC at the two frequencies. The looking time data analysis was conducted on the entire trial duration (28.8 s), whereas the EEG data analysis was performed on the three 9 s epochs created by dividing each trial into smaller segments (see the Data Processing section for details). On average, participants contributed 13.98 trials (*SD* = 0.14) to the looking time data analysis and 41.51 epochs (*SD* = 2.60) to the EEG data analysis.

#### Looking Behavior.

**Figure 5.**
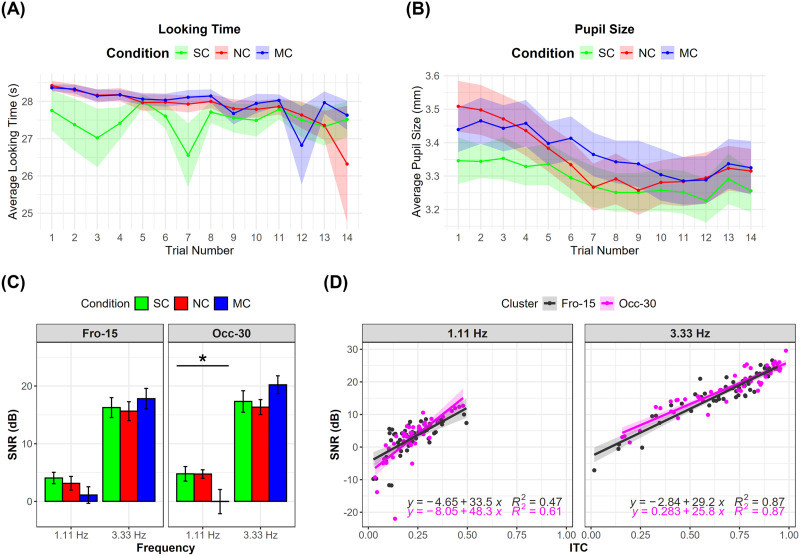
Looking time, pupil size, mean SNR, and the relationship between ITC and SNR at 1.11 Hz (triplet frequency) and 3.33 Hz (image frequency) in the learning trials. *Note*. (A) Looking time (in seconds) and (B) pupil size (in millimeters) plotted across the 14 learning trials for the Single-Category (SC), No-Category (NC), and Mixed-Category (MC) conditions. The shaded regions represent 95% confidence intervals. The statistical analysis revealed no significant differences in looking time between conditions (*p* > .161) and a significant decrease in pupil size across trials (*p* < .001). (C) Mean SNR (in decibels) at 1.11 Hz and 3.33 Hz across experimental conditions for the frontal (Fro-15) and occipital (Occ-30) electrode clusters. Error bars represent standard errors of the mean (SEM). A significant difference in SNR was observed between conditions at 1.11 Hz (*p* < .05), with a stronger effect in the occipital cluster. (D) Scatterplots illustrate the relationship between ITC and SNR across frequencies and clusters. The shaded regions represent 95% confidence intervals for the regression lines. Regression equations and R^2^ values are provided for each cluster. Higher ITC values were associated with higher SNR values across both frequencies.

Participants looked at the stimuli for the entire trial duration (see Figure 5). The average total looking time was 27.76 seconds (*SD* = 1.86), and there were no differences across the 3 conditions. A two-way repeated-measures ANOVA, with Condition as a between-subjects factor and Trial Number as a within-subjects factor, yielded no main effects or interactions (all *F’s* < 1.69, all *p’s* > .161). We also analyzed pupil size, which has previously been associated with arousal (see Bradshaw, 1967; Chang et al., 2025; Janisse, 1973), and found a significant decrease in pupil size across trials, *F*(5.53, 265.59) = 17.72, *p* < .001, *η*_p_^2^ = .27. No other main effects or interactions reached significance (all *F’s* < 1.42, all *p’s* > .161). Overall, these analyses indicate that participants complied with the task demands (i.e., to attend to the stimuli) and that their interest/cognitive effort decreased as the experiment progressed.

#### SNR.

As depicted in Figure 4, SNR peaked at 1.11 Hz (triplet frequency) and at 3.33 Hz (image frequency). To assess whether the observed SNR exceeded chance level, we conducted separate one-sample *t* tests against 0 dB for each of the two electrode clusters and two frequencies (see de Heering & Rossion, 2015; Kabdebon et al., 2022). SNR was significantly different from 0 dB at both frequencies in the frontal cluster (*M* = 2.77, *SD* = 5.06 for 1.11 Hz; *M* = 16.57, *SD* = 6.99 for 3.33 Hz) and in the occipital cluster (*M* = 3.17, *SD* = 6.39 for 1.11 Hz; *M* = 17.97, *SD* = 6.62 for 3.33 Hz; all *t’s*(50) > 3.54, all *p’s* < .001, all Cohen *d*_z_ > 0.50, all BF_10_ > 31.88). Robust SNR at 3.33 Hz confirms reliable stimulus-locked responses (and task engagement), whereas increased SNR at 1.11 Hz, collapsed across conditions, suggests sensitivity to the triplet structure in at least some experimental conditions.

Although SNR peaked at both frequencies, differences emerged between the experimental conditions at the triplet frequency (see Figure 5). A three-way repeated-measures ANOVA, with Condition as a between-subjects factor and Frequency and Cluster as within-subjects factors, revealed a significant main effect of Frequency, *F*(1, 48) = 196.99, *p* < .001, *η*_p_^2^ = .80. The average SNR at 3.33 Hz was 17.27 (*SD* = 6.81) while the average SNR at 1.11 Hz was 2.97 (*SD* = 5.74). This main effect was qualified by a significant Condition × Frequency interaction, *F*(2, 48) = 4.22, *p* = .02, *η*_p_^2^ = .15. No other main effects or interactions reached significance (all *F’s* < 2.52, all *p’s* > .119).

To further clarify the Condition × Frequency interaction, we conducted a one-way ANOVA, with Condition as a between-subjects factor, separately for each frequency. For 3.33 Hz (image frequency), there were no significant differences between the three conditions, *F*(2, 48) = 1.16, *p* = .321, *η*_p_^2^ = .05 (Single-Category: *M* = 16.79, *SD* = 6.67; No-Category: *M* = 16.00, *SD* = 5.23; Mixed-Category: *M* = 19.02, *SD* = 5.98). This absence of differences in stimulus-locked responses at the image frequency suggests that participants were equally attentive across the three conditions.

In contrast to the image frequency, SNR differed across conditions at 1.11 Hz (triplet frequency), *F*(2, 48) = 3.71, *p* = .032, *η*_p_^2^ = .13. Planned contrasts comparing the Mixed-Category condition to the other two conditions showed that SNR was significantly lower in the Mixed-Category condition (*M* = 0.54, *SD* = 6.06) than in the Single-Category condition (*M* = 4.42, *SD* = 3.73), *t*(32) = 2.25, *p* = .031, Cohen *d*_z_ = 0.77, BF_10_ = 2.17, and that it was marginally lower in the Mixed-Category condition than in the No-Category condition (*M* = 3.95, *SD* = 3.34), *t*(32) = 2.03, *p* = .050, Cohen *d*_z_ = 0.70, BF_10_ = 1.55. One-sample *t*-tests comparing the observed SNR at the triplet frequency in each condition to 0 dB chance level revealed significant differences across all clusters and conditions except for the Mixed-Category condition (see Supplementary Materials). In the Mixed-Category condition, category information was misaligned with the triplet structure; hence, the finding that SNR was lowest and did not differ from chance in this condition is consistent with our prediction that such misalignment can hinder learning.

Recall that the pupil data indicated that interest/cognitive effort decreased as the experiment progressed. Therefore, we asked whether neural entrainment was strongest in the first half of the learning trials, when interest/cognitive effort was arguably at its maximum. In addition, we asked whether the alignment between statistical cues for category and exemplar information – as was the case in the Single-Category condition – might have yielded faster encoding of triplets than when those two cues are not aligned – as was the case in the No-Category condition. Accordingly, we analyzed the SNR data for the first seven learning trials. The results were similar to those from all 14 trials except that the main effect of condition did not reach significance at 1.11 Hz (triplet frequency), *F*(2, 48) = 2.07, *p* = .137, *η*_p_^2^ = .08 (Single-Category: *M* = 2.83, *SD* = 2.78; No-Category: *M* = 0.47, *SD* = 7.15; Mixed-Category: *M* = -0.43, *SD* = 3.32). One-sample *t*-tests comparing SNR against 0 dB revealed that SNR at the triplet frequency was significantly above 0 dB in the Single-Category condition but not significantly above 0 dB in the other two conditions. This suggests that learning progressed more rapidly in the Single-Category condition than in the other two conditions (see Supplementary Materials).

#### Relationship Between ITC and SNR.

As expected, given their underlying mathematical similarity, we found that higher ITC values were associated with higher SNR values (see Figure 5). For the 1.11 Hz (triplet frequency), there was a significant correlation between ITC and SNR, *r*(49) = .75, *p* < .001. The regression model was statistically significant, *F*(1, 49) = 63.16, *p* < .001, adjusted *R*^2^ = 0.55, and ITC was a significant predictor of SNR, *t*(49) = 7.95, *p* < .001 (regression equation: SNR = −6.65 + 42.35 * ITC value). Similarly, for 3.33 Hz (image frequency), ITC and SNR were positively correlated, *r*(49) = .94, *p* < .001, and the regression model was statistically significant, *F*(1, 49) = 347.05, *p* < .001, adjusted *R*^2^ = 0.87. ITC significantly predicted SNR, *t*(49) = 18.63, *p* < .001 (regression equation: SNR = −1.21 + 27.34 * ITC value). This pattern of results was observed in both the frontal and the occipital cluster of electrodes. Furthermore, the effect of ITC on SNR was consistent across experimental conditions as revealed by a moderation analysis (see Supplementary Materials).

## DISCUSSION

We used SSVEP to investigate the interaction between category-level and exemplar-level information in visual statistical learning in adults. Specifically, we tested whether category information modulates learning of statistically defined triplets as a function of its alignment versus misalignment with triplet boundaries. To do so, we collected EEG data during the learning phase to obtain a neural index of learning. We then assessed learning behaviorally using a pos*t*-test target-detection task, with reaction time as the dependent measure. This design allowed us to evaluate which of our three predictions best accounted for our findings: (1) no category effect (equivalence across conditions), (2) an additive benefit when category cues align with triplet boundaries (Single-Category > No-Category), and (3) interference when category information is misaligned with triplet structure (No-Category > Mixed-Category).

Reaction time data from the target detection task showed that participants responded faster to the third image in a triplet – compared to earlier image positions in a triplet – in both the Single-Category and No-Category conditions, but not in the Mixed-Category condition. This behavioral response pattern indicates that participants successfully learned the triplets when category-level and exemplar-level information was aligned or did not conflict (i.e., in the Single-Category and No-Category conditions) but that they did not learn them when the two types of information conflicted (i.e., in the Mixed-Category condition). In terms of our predictions, the absence of learning in the Mixed-Category condition supports the interference account (Prediction 3: No-Category > Mixed-Category).

The EEG results mirrored the behavioral findings. They revealed robust neural entrainment at 1.11 Hz (triplet frequency) in the Single-Category condition, weaker but significant entrainment in the No-Category condition, and no significant entrainment in the Mixed-Category condition. Together, these neural entrainment findings provide converging evidence for online sensitivity to triplet structure during the learning phase and indicate interference when exemplar-level and category-level information were misaligned (No-Category > Mixed-Category). Crucially, although the overall SSVEP signal at the image frequency (3.33 Hz) was stronger than at the triplet frequency (1.11 Hz), the image-frequency response did not differ across conditions. This suggests that the differences observed at the triplet frequency were likely due to learning rather than low-level perceptual properties of the stimuli. Furthermore, looking time and pupil size measures collected during the learning phase enabled us to rule out the possibility that the observed condition effects were due to differences in task engagement or compliance.

Overall, the present study advances the existing statistical learning research in four ways discussed below. First, we show that visual statistical learning can be detected with non-invasive EEG (see also Sáringer et al., 2024). This is significant because online measures of visual statistical learning have previously been recorded primarily using the invasive ECoG technique (Henin et al., 2021; Sherman et al., 2022, 2023) and were therefore limited to patients awaiting brain surgery for neurological conditions. By contrast, EEG enables the study of statistical learning in healthy adults and children. In the current study, we not only detected neural entrainment in healthy adults, but consistent with the ECoG literature, we also observed entrainment in the fronto-central and parieto-occipital electrodes. This suggests that the two methodologies capture the same neural response. Furthermore, we extended the ECoG work by providing clear evidence of a reliable f/3 component in a triplet-structured, rather than pair-structured, visual statistical learning paradigm.

Second, EEG-based neural entrainment at the triplet frequency provided an online measure of learning that complemented pos*t*-test reaction times and allowed us to test whether category and statistical cues have additive effects on chunk learning. Our findings show that the category relations among exemplars that define the triplet-based structure affect the robustness of neural entrainment, mirroring prior behavioral findings (Rogers et al., 2021; Wang et al., 2020). Specifically, when all three exemplars within a triplet came from the same category (Single-Category condition), triplet-frequency entrainment was reliable and detectable as early as the first half of the learning phase, consistent with the idea that aligned category- and exemplar-statistics can facilitate learning. In contrast, when category information was misaligned with the triplet structure, such that exemplars from the same category were distributed across different triplets (Mixed-Category condition), triplet-frequency entrainment was no longer evident. This suggests that misaligned category cues can interfere with triplet learning, likely by promoting an alternative organization of the visual sequence (e.g., grouping by category membership or tracking category recurrence). Importantly, learning was preserved when all the exemplars in the sequence came from different categories and there was minimal perceptual overlap between them (No-Category condition). This finding indicates that category information does not impair learning *per se*; rather, the contrast between No-Category (learning present) and Mixed-Category (learning absent) points to misalignment as the critical factor. Overall, these results are consistent with prior work showing that category information can shape statistical learning in some paradigms (e.g., Brady & Oliva, 2008; Emberson & Rubinstein, 2016), particularly in those paradigms that increase reliance on category structure or test generalization beyond trained exemplars. These results also suggest that category cues can either foster or hinder statistical learning depending on whether they converge with, or compete against, statistically defined chunk boundaries.

Third, the current study is unique in the statistical learning domain in that it provides parallel analyses of neural entrainment using the two primary metrics reported in the literature: SNR and ITC. We conducted these analyses because prior studies typically report only one of these measures, often without providing a clear rationale for their choice. For example, ITC is commonly used in statistical learning research (Batterink & Paller, 2017; Henin et al., 2021; Sáringer et al., 2024; Sherman et al., 2022, 2023), whereas SNR is more often used in visual oddball paradigms (Liu-Shuang et al., 2016; Rossion et al., 2015). Although the overall pattern of results was similar across both metrics – possibly due to their shared mathematical foundation – SNR appeared to be slightly more robust (see also Ganea et al., 2025). The convergence across the SNR and ITC measures suggests that the differences across the three conditions at the triplet frequency reflect learning-related brain activity rather than idiosyncrasies of a single analytic measure. Furthermore, this convergence provides empirical justification for reporting both measures in future research. Doing so would enhance methodological transparency and rigor and it would support greater comparability across studies.

Fourth, triplet-frequency entrainment emerged as a robust, response-independent marker of statistical learning in non-invasive EEG. Because this neural index does not require overt responses, it is particularly well suited for infants (Calce et al., 2024; Capparini et al., 2026; Choi et al., 2020), other special populations (Batterink et al., 2025), and study designs that involve extended or repeated exposure. One limitation of our study is that we did not explicitly model learning dynamics within the learning phase. Nonetheless, it is worth noting that prior studies of auditory statistical learning have found that entrainment can track learning over time (Batterink et al., 2024; Batterink & Paller,  2017, 2019; Pinto et al., 2022). In the current study, we examined entrainment both across the full learning phase and within its first half, and we found that entrainment was detectable in the first half in the Single-Category condition (as discussed above). However, a natural next step is to quantify learning trajectories across successive exposure trials to assess how alignment between category information and triplet structure affects the learning rate.

Finally, the current study suggests that future neural entrainment studies of statistical learning may need to optimize the structure and duration of the learning phase. Here, we segmented trials into three 9 s EEG epochs to maximize data retention following automatic movement-artifact rejection. Shortening each trial and increasing the total number of trials may improve the signal quality and streamline the data analysis in future studies. Such design choices will be particularly useful for studies aiming to quantify learning trajectories across the learning phase, especially because behavioral pos*t*-tests provide only an end-point measure and cannot capture moment-to-moment changes in learning. Notably, our findings show that just 6.5 minutes of exposure to structured visual sequences was sufficient for adults to learn the embedded triplet structure in two of the three learning conditions, suggesting that the exposure time required for successful learning may depend on task complexity. Finally, combining measures of neural entrainment with source localization may help determine whether the mechanisms underlying statistical learning are modality-specific or modality-general.

In conclusion, our findings of successful learning of aligned category- and exemplar-level statistics and disrupted learning of misaligned category- and exemplar-level statistics demonstrate that SSVEP-based measures of neural entrainment provide a powerful, non-invasive tool for tracking visual statistical learning in real time. Moreover, our findings show that perceptual organization beyond adjacent and non-adjacent statistics can shape learning. Finally, given that neural entrainment is implicit and that it can track learning on a continuous basis, our findings provide empirical support for the use of neural entrainment in future studies of statistical learning in populations that cannot reliably provide overt responses.

## Acknowledgments

Research in this publication was supported by a grant from the National Institute of Child Health and Human Development of the National Institutes of Health under award number R21HD103931. The content is solely the responsibility of the authors and does not necessarily represent the official views of the National Institutes of Health. The authors declare no conflicts of interest.

## Author Contributions

N.G.: Conceptualization; Investigation; Data Curation; Formal Analysis, Methodology; Writing – original draft. D.G.: Conceptualization; Methodology. R.N.A.: Conceptualization; Writing – original draft. D.J.L.: Conceptualization; Writing – original draft.

## Availability of Data and Materials

This experiment was not pre-registered. Stimuli, data, and custom R and MATLAB scripts are available on the Open Science Framework: https://osf.io/mhr6y/?view_only=8879b507ac9644c384623f1260dc90d8.

## Supplementary Material


