# Supplementary material for: Tracking Visual Statistical Learning with Steady-State Visual Evoked Potentials: Effects of Exemplar and Category Information
Source: Open Mind (Camb). 2026 Jun 17;10:808–26. doi: 10.1162/OPMI.a.358 (PMC13327787; doi:10.1162/OPMI.a.358)
Supplement: Supplementary file 1 [file opmi-10-808-s001.pdf]

Supplementary Materials

Observed SNR versus Chance Level

SM Table 1

One-sample t-tests comparing observed SNR at 1.11 Hz (triplet frequency) to 0 dB noise level across electrode clusters and conditions

**Freq**	**Cluster**	**Condition**	***t*-value**	**df**	***p*-value**	**Sig.**	**Cohen *d***	**BF_{10}**
**1.11 Hz**	**Occ-30**	**SC**	**3.80**	**16**	**.002**	*	**0.92**	**25.60**
		**NC**	**6.39**	**16**	**< .001**	***	**1.55**	**2435.65**
		**MC**	**-0.01**	**16**	**.988**		**< 0.01**	**0.25**
	**Fro-15**	**SC**	**4.13**	**16**	**.001**	***	**1.00**	**46.50**
		**NC**	**2.66**	**16**	**.017**	*	**0.65**	**3.48**
		**MC**	**0.77**	**16**	**.452**		**0.19**	**0.32**

Note. *t*-value = t-test statistic, df = degrees of freedom, *p*-value represents significance level. Sig.: $p < .05$ (*), $p < .001$ (***). Cohen's *d* indicates effect size. BF_{10} represents Bayes Factor evidence strength. Cluster: occipital cluster of electrodes (Occ-30) and frontal cluster of electrodes (Fro-15). Condition: Single-Category (SC), No-Category (NC), and Mixed-Category (MC). The one-sample t-tests were conducted against 0 because 0 dB SNR indicates that the signal and noise levels are equal.

Moderation Analysis: Effect of Condition on the ITC – SNR Relationship

A moderation analysis was conducted using PROCESS Model 1 (Hayes, 2022) to examine whether Condition (Single-Category, No-Category, Mixed-Category) moderated the relationship between ITC and SNR at 1.11 Hz (triplet frequency) and 3.33 Hz (image frequency). Results showed that the main effect of ITC was statistically significant for both 1.11 Hz (β =

44.41, $p < .001$) and 3.33 Hz ($\beta = 27.20$, $p < .001$), indicating a strong positive association between ITC and SNR (see SM Figure 1). However, Condition did not significantly moderate this relationship at either frequency ($p = .77$ for 1.11 Hz, $p = .26$ for 3.33 Hz). The interaction term (ITC × Condition) did not contribute significantly to the model ($\Delta R^2 < 0.001$, $p = .773$ for 1.11 Hz; $\Delta R^2 = 0.003$, $p = .264$ for 3.33 Hz), suggesting that the relationship between ITC and SNR remained stable across conditions.

SM Figure 1

Association between ITC and SNR across conditions at 1.11 Hz (triplet frequency) and 3.33 Hz (image frequency)

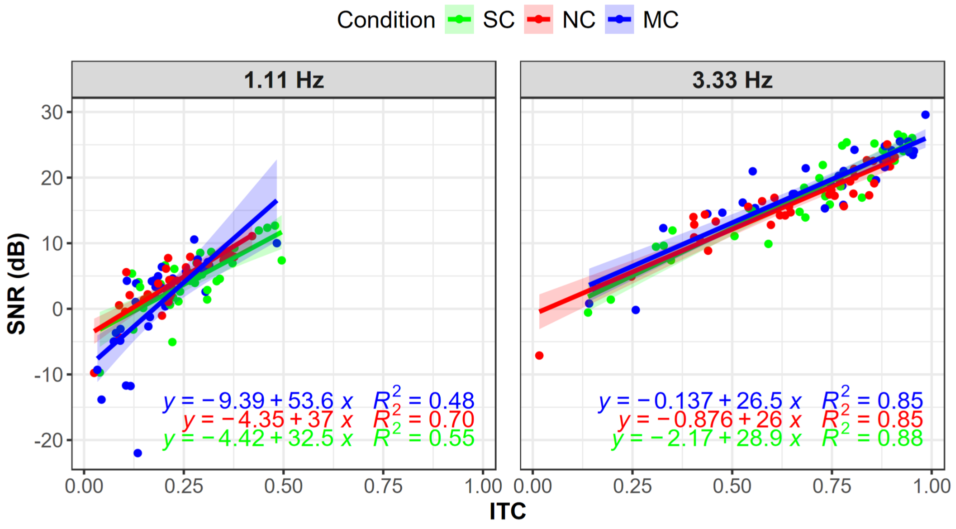

Note. Scatterplots illustrate the relationship between ITC and SNR at 1.11 Hz and 3.33 Hz for different conditions: Single-Category (SC), No-Category (NC), and Mixed-Category (MC). The shaded regions represent 95% confidence intervals for the regression lines. Regression equations and R^2 values are provided for each condition.

Observed ITC versus Chance Level

To assess whether the observed inter-trial coherence (ITC) values at 1.11 Hz (triplet frequency) and 3.33 Hz (image frequency) reflected genuine phase-locking rather than chance fluctuations, we generated a surrogate ITC distribution. Surrogate epochs were extracted with the same duration (9 seconds) as those used in the original analysis, but with randomized time onsets relative to the original data. To avoid overlapping with the original epochs and preserve temporal independence, each surrogate epoch onset was jittered within a window of ±900 ms around the original epoch onset (T_0). Epochs were further constrained to remain non-overlapping with one another. This procedure was performed once per epoch, yielding a single surrogate ITC estimate per frequency of interest (see Kabdebon et al., 2015).

Observed ITC spectra exhibited prominent peaks at 1.11 Hz, 3.33 Hz, and their harmonics (see SM Figure 2), consistent with entrainment to both the triplet and image presentation rates. In contrast, the surrogate ITC spectra showed no clear peaks. To statistically assess these differences, we conducted a 2 × 2 × 2 repeated-measures ANOVA with Cluster (Frontal vs. Occipital), ITC Type (Observed vs. Surrogate), and Frequency (1.11 Hz vs. 3.33 Hz) as within-subjects factors. The analysis revealed a main effect of ITC Type, with Observed ITC ($M = .45$, $SD = .29$) significantly higher than Surrogate ITC ($M = .13$, $SD = .07$), $F(1, 50) = 527.98$, $p < .001$, $\eta_p^2 = .91$. A main effect of Frequency was also observed: ITC was higher at 3.33 Hz ($M = .41$, $SD = .32$) than at 1.11 Hz ($M = .18$, $SD = .10$), $F(1, 50) = 169.63$, $p < .001$, $\eta_p^2 = .77$. These effects were qualified by a significant ITC Type × Frequency interaction, $F(1, 50) = 166.05$, $p < .001$, $\eta_p^2 = .77$. No other main effects or interactions reached significance (all $F < 1.83$, all $p > .182$). To follow up on the interaction, we conducted paired-samples t-tests

comparing Observed and Surrogate ITC at each frequency. At both 1.11 Hz and 3.33 Hz, Observed ITC was significantly higher than Surrogate ITC:

- 1.11 Hz: $M = .23$, $SD = .10$ (Observed) vs. $M = .13$, $SD = .06$ (Surrogate), $t(50) = 8.12$, $p < .001$, Cohen $d_s = 1.49$, $BF_{10} > 100$.
- 3.33 Hz: $M = .68$, $SD = .23$ (Observed) vs. $M = .14$, $SD = .07$ (Surrogate), $t(50) = 15.14$, $p < .001$, Cohen $d_s = 2.66$, $BF_{10} > 100$.

SM Figure 2

Mean ITC peaks across electrode clusters and experimental conditions

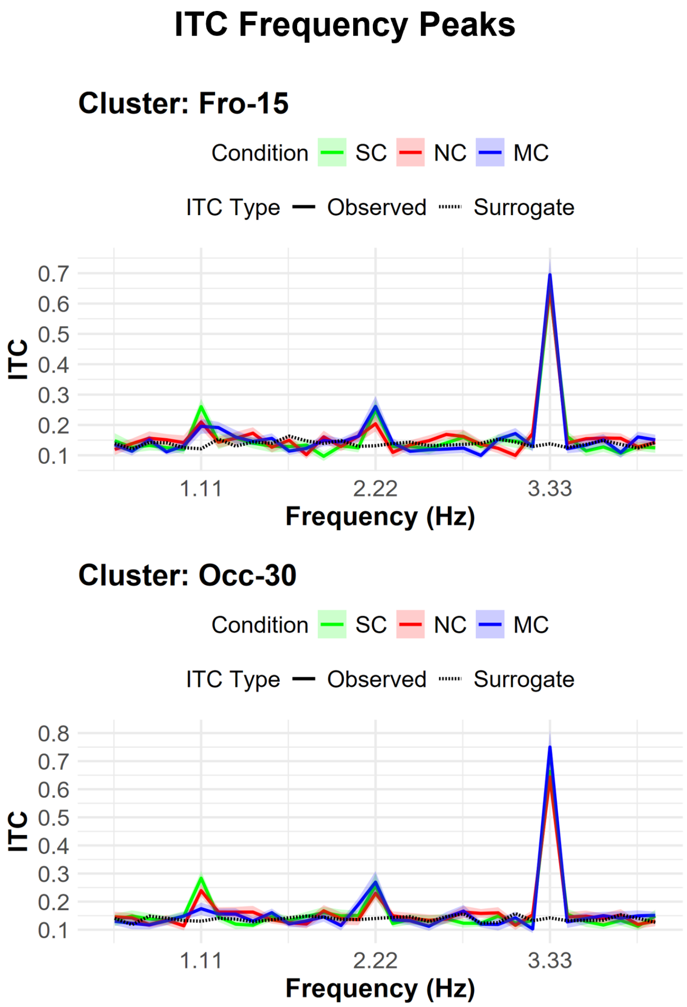

Note. Line plots show the mean inter-trial coherence (ITC) across conditions, with shaded areas representing the standard error of the mean for the frontal electrode cluster (top) and the occipital cluster (bottom). ITC peaked at 1.11 Hz, 3.33 Hz, and their harmonics. The black line depicts the surrogate ITC spectrum. Surrogate ITC was computed by re-segmenting the EEG data into 9-second epochs with onsets jittered within ±900 ms of the original epoch onset (T_0), thereby disrupting temporal alignment with the stimulus while preserving spectral structure. Conditions: Single-Category (SC), No-Category (NC), and Mixed-Category (MC).

Observed ITC across Conditions

Just as with the SNR results, there were condition-related differences in ITC, although the effects were smaller (see SM Figure 3). A three-way mixed ANOVA with Condition as a between-subjects factor and Frequency and Cluster as within-subjects factors revealed a significant main effect of Frequency: ITC was significantly higher at 3.33 Hz ($M = .68$, $SD = .23$) than at 1.11 Hz ($M = .23$, $SD = .10$), $F(1, 48) = 192.69$, $p < .001$, $\eta_p^2 = .80$. The Condition × Frequency interaction was marginally significant, $F(2, 48) = 2.01$, $p = .077$, $\eta_p^2 = .15$. No other main effects or interactions reached significance (all $F < 0.94$, all $p > .19$).

Given the marginal interaction, we conducted follow-up one-way ANOVAs with Condition as a between-subjects factor at each frequency to determine whether ITC differences paralleled those observed for SNR. At 3.33 Hz (image frequency), there were no significant differences in ITC across the three conditions, $F(2, 48) = 0.65$, $p = .525$, $\eta_p^2 = .03$ (*Single-Category*: $M = .66$, $SD = .21$; *No-Category*: $M = .65$, $SD = .19$; *Mixed-Category*: $M = .72$, $SD = .21$). However, at 1.11 Hz (triplet frequency), ITC differed significantly across conditions, $F(2, 48) = 5.22$, $p = .009$, $\eta_p^2 = .18$. We followed up with two planned contrasts comparing the *Mixed-Category* condition to each of the other two:

- *Mixed-Category* vs. *Single-Category*: ITC was significantly lower in the *Mixed-Category* condition ($M = .19$, $SD = .07$) than the *Single-Category* condition ($M = .27$, $SD = .09$), $t(32) = 3.21$, $p = .003$, Cohen $d_z = 1.10$, $BF_{10} = 12.72$.
- *Mixed-Category* vs. *No-Category*: No significant difference between the *Mixed-Category* and the *No-Category* conditions ($M = .22$, $SD = .08$), $t(32) = 1.55$, $p = .131$, Cohen $d_z = 0.53$, $BF_{10} = 0.82$.

SM Figure 3

Mean ITC at 1.11 Hz (triplet frequency) and 3.33 Hz (image frequency) across electrode clusters and experimental conditions

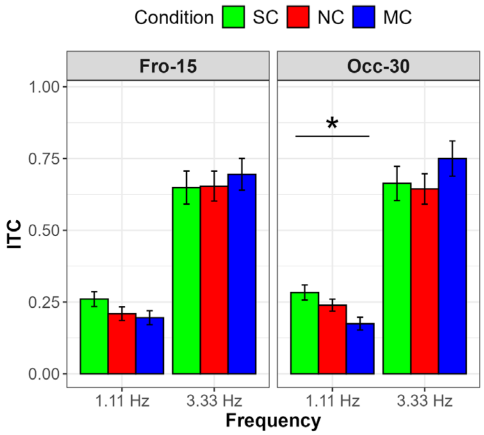

Note. Mean ITC at 1.11 Hz and 3.33 Hz across experimental conditions for the frontal (Fro-15) and occipital (Occ-30) electrode clusters. Error bars represent standard errors of the mean (SEM). A significant difference in ITC was observed between conditions at 1.11 Hz ($p < .05$), with a stronger effect in the occipital cluster. Conditions: Single-Category (SC), No-Category (NC), and Mixed-Category (MC).

First Half of the Learning Phase: Observed SNR

We conducted this analysis on the first seven learning trials to assess whether the effects observed across the entire learning phase emerged early during learning (see SM Figure 4). For 3.33 Hz (image frequency), there were no significant differences between the three conditions, $F(2, 48) = 1.07$, $p = .350$, $\eta_p^2 = .04$ (Single-Category: $M = 13.46$, $SD = 6.87$; No-Category: $M = 13.79$, $SD = 4.50$; Mixed-Category: $M = 16.33$, $SD = 7.04$). Similarly, there were no significant differences between conditions at 1.11 Hz, $F(2, 48) = 2.07$, $p = .137$, $\eta_p^2 = .08$ (Single-Category: $M = 2.83$, $SD = 2.78$; No-Category: $M = 0.47$, $SD = 7.15$; Mixed-Category: $M = -0.43$, $SD =$

3.32). Further one-sample t-tests comparing observed SNR at the triplet frequency with the 0 dB chance level revealed a significant difference only in the Single-Category condition (see SM Table 2).

SM Figure 4

Mean SNR (in decibels) at 1.11 Hz (triplet frequency) and 3.33 Hz (image frequency) in the first half of the learning phase

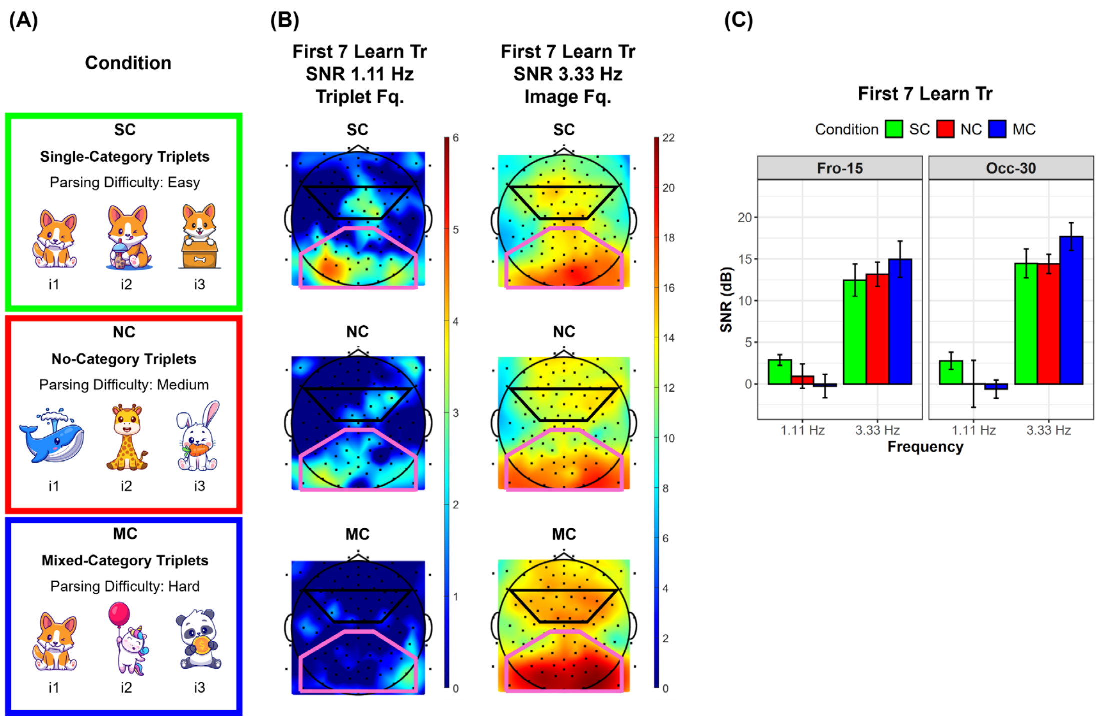

Note. **(A)** Example of triplets presented in each experimental condition. **(B)** Topo plots represent mean SNR at 1.11 Hz (triplet frequency) and 3.33 Hz (image frequency) measured during the first 7 learning trials. SNR was stronger at the image frequency than the triplet frequency. Warmer colors represent higher SNR. **(C)** Mean SNR at 1.11 Hz and 3.33 Hz across experimental conditions for the frontal (Fro-15) and occipital (Occ-30) electrode clusters. Error bars represent the standard error of the mean (SEM). No significant differences between conditions were detected at either frequency. SNR was significantly above the 0 dB chance level at the image frequency across all experimental conditions, and at the triplet frequency only in the Single-Category condition.

SM Table 2

One-sample t-tests comparing observed SNR at 1.11 Hz (triplet frequency) to 0 dB noise level across electrode clusters and conditions in the first half of the learning phase

Freq	Cluster	Condition	*t*-value	df	*p*-value	Sig.	Cohen *d*	BF_{10}
**1.11 Hz**	**Occ-30**	**SC**	**2.68**	**16**	**.017**	*	**0.65**	**3.55**
(First 7 Learn Tr)		**NC**	**0.00**	**16**	**.997**		**0.00**	**0.25**
		**MC**	**-0.57**	**16**	**.578**		**0.14**	**0.29**
	**Fro-15**	**SC**	**4.47**	**16**	**<.001**	***	**1.08**	**85.80**
		**NC**	**0.64**	**16**	**.534**		**0.15**	**0.30**
		**MC**	**-0.18**	**16**	**.863**		**0.04**	**0.25**

Note. *t*-value = t-test statistic, df = degrees of freedom, *p*-value represents significance level. Sig.: $p < .05$ (*), $p < .001$ (***). Cohen's *d* indicates effect size. BF_{10} represents Bayes Factor evidence strength. Cluster: occipital cluster of electrodes (Occ-30) and frontal cluster of electrodes (Fro-15). Condition: Single-Category (SC), No-Category (NC), and Mixed-Category (MC). The one-sample t-tests were conducted against 0 because 0 dB SNR indicates that the signal and noise levels are equal.

First Half of the Learning Phase: Looking Behavior

In the first seven learning trials, participants looked at the stimuli for an average of 27.9 s (SD = 1.55). A two-way repeated-measures ANOVA, with Condition as a between-subjects factor and Trial Number as a within-subjects factor, yielded no main effects or interactions (all F's < 2.81, all p's > .07). An analysis of pupil size revealed a significant decrease across the first seven learning trials, $F(2.78, 133.20) = 15.66$, $p < .001$, $\eta_p^2 = .25$. This main effect was qualified by a significant Condition × Trial Number interaction, $F(5.55, 133.20) = 2.75$, $p = .018$, $\eta_p^2 =$

.10. Planned linear trend analyses indicated significant decreases in pupil size across trials in the No-Category condition, $F(6, 16) = 11.11$, $p < .001$, and the Mixed-Category condition, $F(6, 16) = 4.23$, $p = .010$. However, the linear trend did not reach significance in the Single-Category condition, $F(6, 16) = 1.52$, $p = .234$. Degrees of freedom for repeated-measures effects were Greenhouse–Geisser corrected where necessary. Trend analyses report unadjusted degrees of freedom.

Associations Between Neural Entrainment and Behavioral Responses

To explore associations between neural entrainment during learning (indexed by SNR) and behavioral performance at post-test, we computed a Reaction Time Learning Index (RT_LI) as the difference between reaction time to the first and last images in the triplet (RT_LI = RT_i1 − RT_i3), such that larger values indicate better learning of the triplet structure. We then examined the association between RT_LI and SNR using Pearson correlations in the full sample (collapsed across conditions); results can be seen in SM Figure 5. Next, we tested whether Condition moderated this association using an interaction model (RT_LI × Condition). When simple-slope tests suggested condition-specific associations, we conducted exploratory within-condition linear regressions to better characterize the direction and magnitude of the relationship.

Image Frequency (3.33 Hz). Across all participants, RT_LI was not significantly associated with SNR at 3.33 Hz, $r(49) = -.22$, $p = .128$, 95% CI [−.46, .06], $N = 51$. The RT_LI × Condition interaction was not significant, $F(2, 45) = 0.95$, $p = .393$. Simple-slope analyses indicated a significant negative association in Single-Category, $b = -39.00$, $SE = 17.70$, $t(45) = -2.21$, $p = .033$, whereas slopes were not significant in No-Category, $b = 75.80$, $SE = 102.00$, $t(45) = 0.74$, $p = .463$, or Mixed-Category, $b = 3.45$, $SE = 43.90$, $t(45) = 0.08$, $p = .938$. To better characterize the Single-Category association, we conducted an exploratory regression analysis

within the Single-Category condition, which indicated that RT_LI significantly predicted SNR at 3.33 Hz, $b = -39.00$, $SE = 18.20$, $t(15) = -2.14$, $p = .049$, $R^2 = .23$ (adjusted $R^2 = .18$). Specifically, higher RT_LI was associated with lower SNR at the image frequency in the Single-Category condition. Note that, given the non-significant interaction, this condition-specific result should be interpreted with caution.

Triplet Frequency (1.11 Hz). Across all participants, RT_LI was not significantly associated with SNR at 1.11 Hz, $r(49) = .15$, $p = .302$, 95% CI [−.13, .41], $N = 51$. The RT_LI × Condition interaction was not significant, $F(2, 45) = 2.13$, $p = .131$. Simple-slope analyses were not significant in the Single-Category, $b = -6.85$, $SE = 13.50$, $t(45) = -0.51$, $p = .615$; No-Category, $b = -146.00$, $SE = 78.40$, $t(45) = -1.86$, $p = .070$; or Mixed-Category condition, $b = 28.80$, $SE = 33.60$, $t(45) = 0.86$, $p = .396$.

SM Figure 5

Association between RT Learning Index (RT_LI) and SNR at 1.11 Hz (triplet frequency) and 3.33 Hz (image frequency)

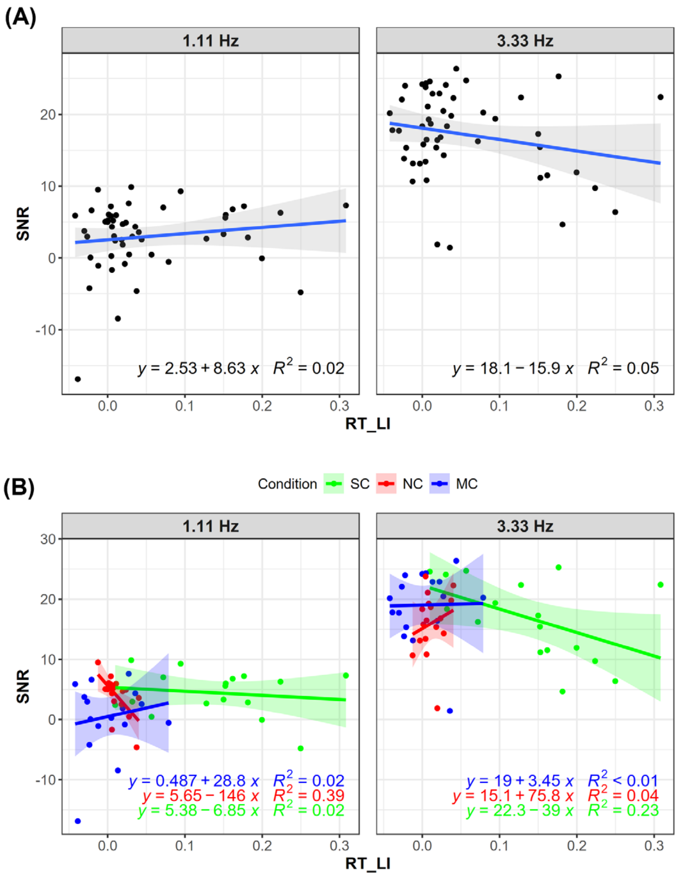

Note. (A) Scatterplots depict the relationship between RT learning index (RT_LI) and SNR collapsed across conditions at 1.11 Hz (Triplet frequency) and 3.33 Hz (Image frequency). (B) The same relationships are shown separately for each condition: Single-Category (SC), No-Category (NC), and Mixed-Category (MC). Points represent individual participants. Solid lines show least-squares regression fits; shaded bands indicate 95% confidence intervals. Regression equations and R^2 values are displayed within each panel. RT_LI = RT_i1 − RT_i3, with larger values reflecting better learning of the triplet structure.
